# The Treatment of Certain Oral Expressions Commonly Designated Pyorrhœa Alveolaris

**Published:** 1901-09

**Authors:** George F. Eames

**Affiliations:** Boston, Mass.


### International Dental Journal.

Vol. XXII. September, 1901. No. 9.

### Original Communications.[1]

### THE TREATMENT OF CERTAIN ORAL EXPRESSIONS COMMONLY DESIGNATED PYORRHŒA ALVEOLARIS.[2]

BY GEORGE F. EAMES, M.D., BOSTON, MASS.

Mr. President and Members of The New York Institute of Stomatology,—The honor implied in your kind invitation to speak to you is deeply appreciated; were this not so, your present speaker would not be in his now precarious position. He finds much relief, however, in the fact that the call of the committee is for remarks on treatment only,—for the simple story of the treatment of various cases in his own office; but it would seem, if a suggestion may be allowed, to be of the greatest practical usefulness if your committee would bring to a meeting of the Institute three or more patients illustrating as many varieties of pyorrhœa, and have every member see the patients for himself.

These cases should be well marked,—not hopeless,—and referred to some member for treatment, and when this is finished, or carried to a certain point, a detailed report may then be given

[1] The editor and publishers are not responsible for the views of authors of papers published in this department, nor for any claim to novelty, or otherwise, that may be made by them. No papers will be received for this department that have appeared in any other journal published in the country.

[2] Read before The New York Institute of Stomatology, May 8, 1901.

to the Institute, the patient again presenting for inspection by the members.

In no other way can you all have the same mental picture of the condition under discussion; therefore in no other way can you talk intelligently about it.

These remarks are not advanced as an elaborate and scholarly essay; the author has no pet theories to announce, makes no pretence to superior skill, has no peculiar or essentially new line of treatment to advocate, but he does urge a more systematic, thorough, and painstaking practice of well-known and established principles of treatment, and he presents a few original suggestions as to the methods by which these principles may be applied.

Prophylaxis is all-important; indeed, in many cases it is absolutely essential to the salvation of the teeth; the term is a broad one, and should include every known means, and every possible effort to prevent disease.

You are familiar with the suggestions of Dr. D. D. Smith, of Philadelphia, as to prophylaxis; the writer is in hearty accord with all that he is doing in this direction, and carries out the treatment as suggested by him, modifying or adding to it as the cases seem to demand.

Dr. Smith argues that there is an increased deposition of tooth-material consequent upon the stimulation or irritation produced by rubbing the surfaces of the teeth with stick and pumice. Obviously, on first thought it would seem well to select only those cases for this treatment in which the teeth need the tissue-building process, and not those which have already been over-stimulated from other causes, such as abrasion, erosion, and calcareous deposits. But these cases necessitate not only the removal of all deposits, but the polishing process as well, and it seems fair to presume that this necessary treatment does not cause increased hypercementosis, or intradentinal deposit, but that the gentle massage applied to the surfaces of the teeth may, after the removal of irritating causes, bring about a cessation of dentinal or cemental development, and an absorption of any soft tissue overgrowth which may exist.

My usual method of procedure in all cases is, first, to spray the mouth with a warm antiseptic solution, using compressed air; this quickly removes any infectious material and enables one to make a better examination.

In all the varieties of this disease, however slight or however great the amount of deposit, this and all other foreign material acting as an irritant are thoroughly removed.

If there is a considerable amount of deposit, and the gums are especially sensitive, an attempt is made to remove a portion of it at the first sitting, as it seems idle to expect any material reduction of the inflammation until this is done.

At the next sitting, the swelling and tenderness being usually reduced, a thorough removal of the deposit may be made.

As a local anæsthetic a five per cent. solution of the oleate of cocaine has failed in my hands to accomplish the desired result. A four per cent. aqueous solution, however, is effective, and the chloroformic mixture suggested by Dr. Cravens gives still better results. A napkin, or its equivalent, is placed so as to protect the parts and check the salivary flow, the pocket is made dry, and the cocaine is either injected or applied by means of floss-silk, or small ropes of cotton placed at the bottom of the pocket and allowed to remain from three to five minutes, while some other part is being treated.

For the removal of the deposit I have devised certain forms of instruments which I will pass around. Very few of those on the market are available for my use. The deposit and all other diseased tissues being removed, the root, in suitable cases, is polished with the orange-wood stick and pumice.

During the operation of removing the deposit the instrument is dipped in a bath containing carbolic acid or other antiseptic solution every time it is removed from the mouth, and the pockets are frequently washed with some antiseptic solution, usually hydrogen peroxide. This is the syringe and the point used for this purpose.

Regarding methods of removing the deposit, no one can tell another exactly how he should operate, or what shape of instrument he should use, to get the best results. I believe that each practitioner will achieve his greatest success by following the method and using the particular instrument which he finds adapted to his own individuality, but this will not prevent him from receiving much benefit from suggestions both as to methods and as to instruments, and it is in this spirit that these little knick-knacks are offered for your consideration. The shapes of these instruments, it seems to me, will suggest at once to you the various uses to which they may be put, therefore a description of their use will not be necessary.

When the pockets have been thoroughly cleansed and the accessible portion of the root polished, a mouth-bath is given. I will pass around the instruments which I use for this purpose; they are connected with the hot and cold water faucets by the ordinary tubing, with the exception of a relief cock for the purpose of testing the temperature of the water and for turning it on and off from the mouth. The water is made as hot as can well be borne in the mouth, and the flushing process is maintained for about three minutes. Following the mouth-bath the antiseptic paste is injected into the pockets by means of the syringe, which I will pass around. The needle is gently pushed to the bottom of the pocket and the screw turned; the plastic material is forced out until it appears at the margin of the gum. A hypodermic needle attached to this syringe may be carried through the gum to inaccessible pockets when necessary. The instruments for the removal of the deposit, the syringe and points for injecting plastics into the pockets and also between the teeth for the prevention of decay, and the mouth-douches are presented to The New York Institute of Stomatology for what they are worth.

The object of the plastic preparation is obvious. It is of such a degree of plasticity that in those large pockets which do not perfectly fill with blood-clots it keeps the pocket free from infection, is soothing to the tissues, and remains for a long time. In shallow pockets and spaces between the teeth I have found that this plastic material can be placed very effectively with the ball of the finger. The prescription for this preparation is as follows: Vaseline, one ounce; white wax, a half-ounce; hydronaphthol or aristol, fifteen grains; menthol, three grains.

The next step in the treatment is the application of a remedy recommended by your worthy ex-president, Dr. E. A. Bogue. It is prepared by dissolving zinc sulphate to saturation in cold water; put one ounce of potassium iodide in two ounces of water, adding as much iodine in crystals as it will take up; then take equal parts of the two solutions and put them together. This solution has many excellent properties, and I often use it at the beginning of treatment, instead of cocaine.

Whatever the part played by bacteria, the treatment is intended to combat their growth as well as their effects.

Massage has long been recognized as an effective means of bringing about an absorption of overgrown tissue, and in those spongy and enlarged conditions of the gum-tissue may be employed, I think, to advantage. The patient is directed to smear the ends of two or three fingers with vaseline one ounce and menthol two grains, and rub the gums surrounding all the teeth for five minutes, twice daily.

In addition to the ordinary bristle tooth-brush, of the best shape at command, I have adapted a brush with rubber bristles for the purpose of brushing the teeth, and especially the gums. The rubber brush appeals to me as having a better effect on the soft tissues, and it is my belief that the patient is more likely to use it on the gums than the brush with hair bristles.

The importance of correcting malocclusion, of supporting loose teeth by fixation, and of inserting bridges between progressively inclining teeth, need hardly be emphasized before this society.

The replantation of teeth around which the disease has progressed to a great extent offers its temptations. It savors of clean surgical work, and there is a good prospect of bettering the condition of the tooth, but we have no assurance of permanency. Replantation is usually delayed until the disease is advanced beyond a hopeful prognosis, it is true, but under much better conditions the majority of teeth are lost at the end of twelve or fifteen years. In other words, when a tooth is so slightly affected by this disease as to offer good hopes in replantation, we may well consider it a promising case under the ordinary treatment *in situ*.

Replantation has its place, however, for in advanced cases the usefulness of a tooth may often be prolonged by replantation and fixation to another tooth. Mouth-breathing undoubtedly has always a deleterious influence, and especially in pyorrhœal cases; the remedy for this trouble should be applied. Teeth having devitalized pulps should certainly receive treatment.

The thorough sanitation of the oral cavity brought about in the treatment of pyorrhœa alveolaris has more than a local effect, for the swallowing of infectious material can do nothing but undermine the general health, and this in turn reacts locally in the mouth. We are thus imperceptibly led to consider the relation of constitutional conditions to local ones as manifested in the mouth.

It is fair to assume that many persons have lived the allotted time of life with an inherited predisposition to tuberculosis simply because a wisely selected mode of living was sufficient to prevent a local outbreak. Likewise, it is also fair to assume that an inherited or an acquired predisposition, with a tendency to a local expression in the mouth in some forms of so-called pyorrhœa, may be kept from doing serious damage to the oral tissues by thorough local and constitutional treatment, and it pays to do this even though it be at the price of "eternal vigilance."

We may institute such thorough local treatment, thus removing all the weak or susceptible points, that the constitutional factor cannot show itself in any of the oral tissues; but the most thorough and scientific constitutional treatment cannot effect a cure while there are filth pockets existing in the mouth. Therefore, a cessation of symptoms is possible by means of intelligent local treatment, but not possible by the most thorough constitutional treatment if local treatment is neglected. In all cases which do not yield to local treatment an examination of the saliva, blood, and urine is made, supplementing, of course, a general examination of the body.

In acute cases to which the term phagedenic pericementitis is applied, and in which there is severe pain, with swelling and looseness of the tooth, I have invariably found rheumatic symptoms. In these cases only the mildest local treatment is applied, consisting of antiseptic injections and washes, and pronounced general treatment calculated to raise the alkalinity of the blood, and to eliminate effete products.

There are many other cases presenting with pain and tenderness in the region of the roots of the teeth for which no local cause can be assigned. Such cases have always responded to constitutional treatment. In all these cases there have been swollen joints, neuralgia, and other symptoms of uric acid poisoning. In speaking of uric acid I do not mean to convey the impression that uric acid always acts as a poison, for, indeed, it exists in the blood normally in a small quantity. A brief allusion to the patient who has come before you may best illustrate this point.

This patient presented about a month ago with turbid and swollen gums containing pus, undoubtedly due to the large light or yellow-colored salivary deposit. He complained of a very disagreeable taste in the mouth; otherwise, he claimed to be in perfect health; no constitutional derangement could be detected from a most careful physical examination.

Having so far as possible eliminated the local causes, with the result of improving the condition of the gums and checking the flow of pus, but not the deposition of lime-salts about the teeth, an analysis of the saliva, urine, and blood was made.

The reaction of the saliva was alkaline; specific gravity, 1006. Total alkalinity equalled 0.0351 per cent. $Na_2Co_3$.

According to the few tables in existence the normal saliva has an alkalinity of about 0.08 per cent. $Na_2Co_3$, with a specific gravity of 1003 to 1006.

The saliva in this case, therefore, contains a large amount of solids and has a low alkalinity.

These facts tend to confirm the supposition indicated by the pathological condition found in this patient's mouth,—viz., that the saliva contained an excessive amount of calcium salts, and though the low alkalinity might serve as a partial cause for the separation of these salts from solution, this cannot be definitely settled until the normal variation of the lime contents of the saliva has been determined.

The supposition may be held, therefore, that there is in this case either an excess of lime or an abnormal condition favoring deposit. The facts seem to suggest an excess of lime-salts rather than an abnormal condition favoring deposit.

The examination of the blood in this case showed it to be practically normal. The urine was of a strongly acid reaction, containing calcic oxalate crystals, giving evidence of a storing up in the body of the normal products of metabolism, especially the xanthine or uric acid group of products. The totals of urea, chlorine, phosphoric acid, and uric acid are all lower than normal.

Treatment will be given to increase the elimination of solids, especially of the uric acid and allied substances; for example, phosphate of soda, fifteen grains t. i. d., and bicarbonate of soda, a salt-spoonful twice daily. The oxaluria gives evidence also, probably, of the same faulty metabolic process, and would yield to the same treatment. The diet should be regulated so as to avoid tomatoes, onions, and an excess of starchy or sweet foods, and the patient should drink three pints of distilled water daily.

